# Investigation on pretreatment method for the determination of rare earth elements in polymetallic mineral samples in Pan-Xi region by ICP-MS

**DOI:** 10.1371/journal.pone.0305559

**Published:** 2024-06-14

**Authors:** Daolu Bu, Yang Ding, Haichuan Lu, Shunxiang Wang, Linyang Shuai, Xiang Xia, Cang Gong

**Affiliations:** 1 Research Center of Applied Geology of China Geological Survey, Chengdu, China; 2 Key Laboratory of Natural Resource Coupling Process and Effects, Beijing, China; Center for Research and Technology Transfer, VIET NAM

## Abstract

The polymetallic mineral samples in Pan-Xi region are rich in rare earth resources, and exploring an efficient and accurate analysis method is of great significance for their comprehensive utilization. In this study, the samples were decomposed by three methods, namely closed acid dissolution, open acid dissolution with five acid and alkali fusion with sodium peroxide, and the 15 REE were determined by inductively coupled plasma mass spectrometry (ICP-MS) with Rh and Re as internal standard correction elements. The comparative experiments were conducted using standard substances, and the results showed: (1) The detection limit of closed acid dissolution method was low with relative standard deviation (RSD) ranging from 2.51% to 9.56% and the accuracy of method (Δlg*C*) ranging from 0.006 to 0.073, while the sample processing was long, and the results of some REE were low. (2) The RSD of open acid dissolution with five acid method ranged from 1.93% to 7.96%, and Δlg*C* ranged from 0.004 to 0.045 with low results of the determination results of REE. (3) The alkali fusion with sodium peroxide method eliminated the influence of matrix effects by selecting instrument optimization, sample dilution, appropriate internal standard elements, etc. The RSD ranged from 1.24% to 6.49%, and Δlg*C* ranged from 0.001 to 0.032. In conclusion, alkali fusion with sodium peroxide method has a fast analysis process, complete sample dissolution, and the accuracy and precision of test results can meet the requirements of specification (DZ/T0011-2015), which is most suitable for the analysis of REE in polymetallic mineral samples from Pan-Xi region.

## Introduction

The Mianning-Dechang rare earth mineralization belt in Panzhihua-Xichang (Pan-Xi) region of Sichuan province is one of the three major rare earth resource bases in China, and the identified resources/reserves rank second, mainly consisting of hard rock type light rare earth ores. With the extensive exploitation and utilization of these ores, high-grade ores have gradually decreased, and the comprehensive development and utilization of low-grade and complex polymetallic ores have been receiving increasing attention [1, 2]. The rapid and accurate determination of rare earth element content in polymetallic ores in Pan-Xi region is of great significance for the study of metallogenic law of rare earth resources, as well as the optimization of prospecting targets in the region. However, there are few reports on the research of its determination methods nowadays.

At present, the widely used methods for the determination of REE in polymetallic ore samples include X-ray fluorescence spectroscopy (XRF) [3, 4], inductively coupled plasma spectroscopy (ICP-AES) [5–7] and inductively coupled plasma mass spectrometry (ICP-MS) [8–10]. Among them, the XRF method for determining REE requires a large amount of standard substances to establish a standard curve, and there are few ore standard substances that match its matrix, moreover, the spectrum lines of rare earth element overlap severely [11]. Compared with XRF, ICP-AES and ICP-MS are currently the main analysis and testing methods for REE. However, ICP-MS is widely used for determination of REE in geological samples due to its advantages of low detection limit, wide linear range, and relatively less interference [11–14]. The pre-treatment methods for ICP-MS determination of REE mainly include closed acid dissolution method [15, 16], open acid dissolution method [10, 16, 17], alkali melting method [18, 19], and microwave digestion method [20]. Among them, microwave digestion method is not suitable for simultaneously processing large batches of samples due to the small number of samples processed in a single process [21]. Based on the above considerations, this article focuses on the characteristics of REE being rich in polymetallic ore samples of Pan-Xi region. ICP-MS method was used to study the effects of closed acid dissolution method, open acid dissolution with five acid method and alkali fusion with sodium peroxide method on the analysis and determination results through comparative experiments. This provides a scientific, accurate, and efficient analysis method for the determination of REE in polymetallic ore samples in Pan-Xi region.

## Material and methods

### Instruments and working parameters

The main instrument used in the experiment is X-Series II inductively coupled plasma mass spectrometer (ThermoFisher, USA), with main operating parameters: incident power of 1300 W, atomization gas flow rate of 0.9 L·min^-1^, cooling gas flow rate of 13 L·min^-1^, auxiliary gas flow rate of 0.8 L·min^-1^, truncated cone aperture of 0.7 mm, sampling depth of 150 mm, oxide yield of below 3%, double charge yield of below 2%, scanning frequency of 55 times, and scanning method of peak jumping.

### Standard solution and main reagents

Single element standard reserve solution for 17 REE (La, Ce, Pr, Nd, Pm, Sm, Eu, Gd, Tb, Dy, Ho, Er, Tm, Yb, Lu, Sc and Y), ^103^Rh and ^185^Re (National Nonferrous Metals and Electronic Materials Analysis and Testing Center): (1) mass concentration of each element is 1000 μg/mL; (2) the standard curve series is prepared by gradually diluting with 3% nitric acid. ICP-MS tuning solution Tune A (Li, Co, In, Ba, Bi, U and other elements): mass concentration is all 1 ng/mL.

Hydrochloric acid, nitric acid, hydrofluoric acid, sulfuric acid, and perchloric acid are all of superior purity (Chengdu Cologne Chemical Co., Ltd.). The experimental water is high-purity water with a resistivity greater than 18.25 MΩ·cm. The experimental vessels were soaked in 10% aqua regia (volume fraction) for more than 48 h, washed with pure water 3~5 times, and then dried for later use.

Samples used in the experiment: National standard substances GBW07309, GBW07301a, GBW07408, GBW07430, GBW07451,GBW07107 for water system, soil, and rock, as well as rare earth ore standard substances GBW07890, GBW07891, GBW07892, and GBW07893 (raw materials were collected from natural ores which came from Miaoya in Zhushan, Hubei, Tianbao in Zhuxi, Hubei, and Maoniuping in Sichuan, respectively).

### Sample decomposition methods

#### (1) Closed acid dissolution

Accurately weigh 0.0500 g of sample dried at 110°C and place it in PTFE inner tank of closed dissolution device. Add 1 mL of hydrofluoric acid and 1 mL of nitric acid, and place it in a 190°C oven for 48 h of insulation. After cooling, open the container and place it on a 180°C electric heating plate to evaporate and dry. Add 1 mL nitric acid and evaporate to near dryness. Repeat this step twice. Add 2 mL aqua regia (1+1), place it in an oven at 140°C for 6 h, take it out and cool it, then open it, transfer the solution into a 50 mL volumetric flask, make up to constant volume with 3% HNO_3_ (volume fraction), shake well, and put it on the machine for testing.

#### (2) Open acid dissolution with five acids

Accurately weigh 0.2500 g of sample dried at 110°C and place it in a 50 mL PTFE crucible. Wet it with a few drops of water, add 5 mL of hydrochloric acid, and place it on an electric heating plate to maintain a temperature of 200°C close to evaporation. Add 5 mL of nitric acid, 10 mL of hydrofluoric acid, 2 mL of perchloric acid, and 3 mL of 50% (volume fraction) sulfuric acid and heat up to 260°C. When the white smoke of perchloric acid is exhausted, cool the electric heating plate to 180°C and add 10 mL of aqua regia to extract salts. After the small bubbles have been exhausted, add 5 mL of ultrapure water, heat for 5 min, remove and cool to room temperature. Transfer to a 25 mL volumetric flask, make up to volume, shake well, and let it stand. Transfer 1 mL of the upper clear liquid into a 10 mL colorimetric tube, make up to constant volume with 3% HNO_3_ (volume fraction), shake well, and put it on the machine for testing.

#### (3) Alkali fusion with sodium peroxide

Accurately weigh 0.2500 g of sample dried at 110°C and place it in a corundum crucible. Add 1.50 g of sodium peroxide and mix well, then cover with 0.50 g of sodium peroxide. Put the crucible into a muffle furnace that has been preheated to 700°C, melt for 18 min, and take it out for cooling. Place the cooled crucible in a beaker containing 50 mL of water, and heat it on an electric heating plate to a slight boil. When the melting block falls off, leave it overnight. Slow filter paper (No.42) is used for filtration, the resulting precipitate is washed with 2% sodium hydroxide solution for 4~5 times, then the precipitate is dissolved with 1:1 hot hydrochloric acid solution and transferred to a 50 mL volumetric flask. The sample is diluted 10 times and transferred to colorimetric tube for measurement.

## Results and discussions

### Selection of internal standard elements and correction of matrix interference

The internal standard calibration method is one of the quantitative calibration methods commonly used in many analytical techniques. The internal standard method can not only effectively monitor and correct the short-term and long-term drift of signals, but also correct the matrix effect caused by samples [22]. The selection of internal standard elements is particularly important, and its selection principle is that tested solution does not contain selected internal standard elements, interference factors of internal standard elements are minimized, and mass number of internal standard elements is matched with the tested elements as much as possible. Generally speaking, the isotopes of internal standard elements that basically meet the above conditions are ^115^In, ^103^Rh and ^187^Re. However, research by Qianni Men et al. [10] pointed out that the content of ^115^In in sample is sometimes high, and ^115^In is severely affected by ^115^Cd interference. The content of ^103^Rh and ^187^Re in geochemical samples is extremely low.Through the national first-class standard material GBW07451 experiments, the results are shown in Table 1,and it is concluded that this paper chooses ^103^Rh and ^187^Re as internal standard elements for determination of all REE by ICP-MS, which can effectively compensate for errors caused by matrix effects and achieve good experimental results.

**Table 1:** Correction effects of internal standard elements on determination results.

REE	internal standard elements	Certified value (μg.g^-1^)	No internal standard correction		After internal standard correction	
			Measured value (μg.g^-1^)	Δlg*C*	Measured value (μg.g^-1^)	Δlg*C*
^89^Y	^103^Rh	25	22.8	0.039	24.73	0.005
^139^La	^103^Rh	44	39.4	0.048	43.38	0.006
^140^Ce	^103^Rh	81	91.4	0.053	80.1	0.005
^141^Pr	^103^Rh	9.4	8.50	0.044	9.14	0.012
^146^Nd	^187^Re	35	30.3	0.062	34.1	0.011
^147^Sm	^187^Re	6.1	7.54	0.092	6.30	0.014
^153^Eu	^187^Re	1.3	1.09	0.078	1.24	0.020
^157^Gd	^187^Re	5.3	6.43	0.084	5.24	0.005
^159^Tb	^187^Re	0.85	0.738	0.062	0.870	0.010
^163^Dy	^187^Re	4.6	5.27	0.059	4.69	0.009
^165^Ho	^187^Re	0.93	1.03	0.046	0.904	0.013
^167^Er	^187^Re	2.6	2.45	0.026	2.65	0.009
^169^Tm	^187^Re	0.43	0.402	0.030	0.440	0.010
^172^Yb	^187^Re	2.8	2.57	0.037	2.70	0.016
^175^Lu	^187^Re	0.43	0.463	0.032	0.444	0.013

Siewers [23] conducted a detailed study on matrix interference caused by total dissolved solids (TDS) in solutions determined by ICP-MS. When the TDS is 500 μg/mL, the analytical signals of measured elements drift significantly within a short period of time, and it is generally required that the TDS should be less than 0.1%. However, due to the extremely complex background of geological samples, differences in the proportion of elements in sample can also cause matrix effects. This matrix effect can be effectively reduced through instrument optimization and sample dilution [24]. In this paper, ^103^Rh and ^187^Re were selected as internal standard elements to monitor and correct the signal drift, and the national standard substances were used to prepare a solution along with sample, which was used to draw a standard curve to test sample, so as to eliminate matrix effects and background bias, and significantly to improve test results.

### Mass spectrometry interference correction

For determination of REE in geological samples by ICP-MS, each rare earth element has at least one isotope that is not interfered by congruent isotopes, and the sensitivity is relatively high from ^139^La to ^175^Lu, which is subjected to relatively little interference. Among them, Y, La, Ce, and Pr all have only one isotope suitable for ICP-MS determination, and the mass spectrometry interference is very small, so interference correction is not necessary. Three isotopes of Nd and two isotopes of Sm can be used for ICP-MS determination, the measurement results of each isotope are very close to the standard values and do not require interference correction. The isotope with the highest abundance is selected for measurement. The isotopes selected for the determination of each rare earth element were ^89^Y, ^139^La, ^140^Ce, ^141^Pr, ^146^Nd, ^147^Sm, ^153^Eu, ^157^Gd, ^159^Tb, ^163^Dy, ^165^Ho, ^167^Er, ^169^Tm, ^172^Yb, and ^175^Lu. However, the interference of oxides, such as Ba oxide on Eu and light rare earth element oxide on heavy REE, cannot be ignored, especially when the content of elements that can produce oxides in analysis sample is high and the content of interfering elements is low. Among them, ^153^Eu needs to be corrected with ^135^Ba^16^O for interference, ^157^Gd needs to be corrected with ^141^Pr^16^O for interference, and ^159^Tb needs to be corrected with ^143^Nd^16^O for interference, which must be corrected using mathematical methods. The interference correction formula is:

$$\rho M\left(\text{t}\right)=\rho M\left(\text{m}\right)-k\times \rho N\left(\text{m}\right)$$

Where, *k* is the interference correction coefficient of the interfering element, *k* = *ρM*(eq)/*ρN*(m), *ρM*(t) is the true concentration of element *M* after correction, *ρM*(m) is the apparent concentration of *M* measured, *ρN*(m) is the concentration of interfering element N in the measured sample solution, *ρM*(eq) is the equivalent concentration value contributed by the pure interfering element *N* solution at *M*. The corresponding interference ion correction coefficients are shown in Table 2.

**Table 2:** Test isotopes and interference ion correction coefficient.

Isotope	Interfering ions	Correction coefficient(%)
^153^Eu	^135^Ba^16^O	0.038
^157^Gd	^141^Pr^16^O	0.016
^159^Tb	^143^Nd^16^O	0.27

### Evaluation of analytical methods

#### Selection of decomposition methods

The national first-level standard material GBW07408 and rare earth ore standard material GBW07890 were selected and treated according to the decomposition method mentioned in the Sample decomposition methods section. The national first- level standard reference material was used to make a standard curve for the determination of REE, each sample was determined 12 times in parallel. The results are shown in Table 3, it can be seen from the analysis results that closed acid dissolution method requires less acid to decompose the sample in a closed environment, resulting in less environmental pollution and low detection limit. However, the sample weighing amount is only 0.0500 g, which is easily affected by the small amount and uneven sample size. Additionally, incomplete redissolution of certain REE due to insufficient hydrofluoric acid or excessive evaporation results in low determination values of some heavy REE [13]. The open acid dissolution with five acid method is used to decompose, although sulfuric acid with high boiling point is added to increase the temperature of acid driving process and to reduce the formation of insoluble rare earth fluoride precipitation by hydrofluoric acid and REE, there is still a very small amount of insoluble residue after dissolution. If the sample is not completely dissolved or some rare earths are co-precipitated by sulfate precipitation, it will lead to low analytical results [11]. Moreover, there are many kinds of acid, which will introduce the interference of polyatomic ions, resulting in the decrease of atomization efficiency. The method of alkali fusion with sodium peroxide is a traditional classical method for decomposing samples, which can decompose all kinds of minerals containing REE [25]. The sample solution after alkaline decomposition of sodium peroxide is clear and transparent, meanwhile without residue phenomenon. The sample test results are accurate, indicating that this method has a good decomposition effect on polymetallic ore samples of Pan-Xi region.

**Table 3:** Determination results of REE in GBW07408 and GBW07890 with three sample decomposition methods by ICP-MS (μg.g^-1^).

REE	GBW07408				GBW07890			
	Certified value	closed acid dissolution method	open acid dissolution with five acid method	alkali fusion with sodium peroxide method	Certified value	closed acid dissolution method	open acid dissolution with five acid method	alkali fusion with sodium peroxide method
^89^Y	26	25.62	25.67	26.33	97.7	94.5	95.9	98.6
^139^La	36	35.58	35.44	36.41	6580	6503	6487	6609
^140^Ce	66	64.87	64.18	67.27	9500	9426	9395	9452
^141^Pr	8.3	8.2	8.16	8.24	824	807	802	819
^146^Nd	32	31.42	31.30	31.55	2320	2263	2275	2341
^147^Sm	5.9	5.83	5.79	5.86	219	210	207	218
^153^Eu	1.2	1.14	1.15	1.22	41.3	39.9	40.3	40.8
^157^Gd	5.4	5.25	5.29	5.38	74.7	72.6	73.5	75.3
^159^Tb	0.89	0.87	0.86	0.89	5.27	5.14	5.17	5.23
^163^Dy	4.8	4.63	4.66	4.91	25.4	24.77	25.01	25.78
^165^Ho	0.97	0.92	0.94	0.98	3.99	3.87	4.06	3.94
^167^Er	2.8	2.63	2.67	2.88	8.71	8.59	8.62	8.75
^169^Tm	0.46	0.41	0.43	0.46	1.16	1.13	1.15	1.15
^172^Yb	2.8	2.74	2.75	2.78	7.43	7.25	7.31	7.48
^175^Lu	0.43	0.40	0.41	0.44	1.11	1.07	1.09	1.1

#### Method detection limit

Ten blank experiments of sample were carried out by using the above three decomposition methods in this paper, and three times standard deviation of determination results was the detection limit of this method. The detection limits of REE analyzed by above three methods are shown in Table 4. It can be seen that the detection limits of REE are lower than the requirements of geochemical survey specification (1:50000) (DZ/T0011-2015). Among them, the detection limit of closed acid dissolution method is obviously lower than that of the other two methods. This is because in a high-pressure closed acid dissolution environment, acid does not evaporate, the amount of acid used is small, and the sample is less contaminated, ensuring a low blank [26].

**Table 4:** Detection limit of the method.

REE	Detection limit (μg.g^-1^)			REE	Detection limit (μg.g^-1^)		
	closed acid dissolution method	open acid dissolution with five acid method	alkali fusion with sodium peroxide method		closed acid dissolution method	open acid dissolution with five acid method	alkali fusion with sodium peroxide method
^89^Y	0.0057	0.032	0.026	^159^Tb	0.0030	0.026	0.029
^139^La	0.0079	0.035	0.031	^163^Dy	0.0024	0.016	0.015
^140^Ce	0.0081	0.050	0.043	^165^Ho	0.0047	0.038	0.031
^141^Pr	0.0027	0.036	0.027	^167^Er	0.0039	0.011	0.012
^146^Nd	0.0036	0.034	0.038	^169^Tm	0.0019	0.039	0.046
^147^Sm	0.0031	0.028	0.022	^172^Yb	0.0046	0.027	0.023
^153^Eu	0.0044	0.019	0.013	^175^Lu	0.0035	0.046	0.038
^157^Gd	0.0034	0.045	0.037	-	-	-	-

#### Method precision and accuracy

Soil standard GBW07107, rare earth standards GBW07891 and GBW07893 were selected, and the samples were processed according to the three decomposition methods mentioned in the Sample decomposition methods section. Each sample was measured 12 times in parallel for each method, average results were taken, and RSD and Δlg*C* of each method were calculated. The results are shown in Table 5.

**Table 5:** Precision and accuracy tests of the three methods.

REE	standards	Certified value μg.g^-1^	closed acid dissolution method			open acid dissolution with five acid method			alkali fusion with sodium peroxide method		
			Average value μg.g^-1^	RSD %	Δlg*C*	Average value μg.g^-1^	RSD %	Δlg*C*	Average value μg.g^-1^	RSD %	Δlg*C*
^89^Y	GBW07107	26	24.8	4.27	0.021	25.5	2.36	0.008	26.3	1.24	0.005
	GBW07891	113	103	3.94	0.040	109	3.27	0.016	115	3.35	0.008
	GBW07893	133	125	5.45	0.027	127	2.98	0.020	129	2.14	0.013
^139^La	GBW07107	62	62.8	3.48	0.006	62.6	3.37	0.004	61.5	1.45	0.004
	GBW07891	365	351	2.51	0.017	379	2.78	0.016	378	3.70	0.015
	GBW07893	10400	10146	4.59	0.011	10251	4.05	0.006	10276	4.82	0.005
^140^Ce	GBW07107	109	115	3.32	0.023	113	2.76	0.016	116	2.79	0.027
	GBW07891	647	618	4.61	0.020	624	3.84	0.016	629	3.46	0.012
	GBW07893	15000	14684	4.57	0.009	14769	5.72	0.007	14856	4.14	0.004
^141^Pr	GBW07107	13.6	13.4	3.73	0.006	13.3	3.95	0.010	13.5	4.26	0.003
	GBW07891	67.2	64.2	2.99	0.020	64.5	4.27	0.018	64.9	3.98	0.015
	GBW07893	1220	1158	3.65	0.023	1176	2.79	0.016	1193	2.59	0.010
^146^Nd	GBW07107	48	46.9	3.32	0.010	47.4	3.37	0.005	48.8	3.80	0.007
	GBW07891	216	203	3.86	0.027	208	1.93	0.016	210	3.66	0.012
	GBW07893	3390	3326	4.67	0.008	3305	3.45	0.011	3328	2.95	0.008
^147^Sm	GBW07107	8.4	8.19	2.92	0.011	8.24	3.73	0.008	8.31	3.06	0.005
	GBW07891	30.9	29.2	4.85	0.025	31.2	2.96	0.004	31.4	4.61	0.007
	GBW07893	313	289	5.63	0.035	282	5.05	0.045	296	6.02	0.024
^153^Eu	GBW07107	1.7	1.66	3.86	0.010	1.65	3.62	0.013	1.73	2.75	0.008
	GBW07891	4.4	4.49	5.03	0.009	4.53	4.37	0.013	4.52	4.08	0.012
	GBW07893	58.9	57.4	4.37	0.011	57.6	2.90	0.010	57.9	3.85	0.007
^157^Gd	GBW07107	6.7	6.38	6.55	0.021	6.43	4.17	0.018	6.48	3.92	0.014
	GBW07891	23.7	22.9	4.37	0.015	23.2	3.79	0.009	24.1	2.46	0.007
	GBW07893	116	105	4.26	0.043	109	4.62	0.027	121	4.37	0.018
^159^Tb	GBW07107	1.02	0.99	6.69	0.013	0.97	5.19	0.022	1.01	5.16	0.004
	GBW07891	3.41	3.26	4.82	0.020	3.35	3.58	0.008	3.39	2.99	0.003
	GBW07893	7.06	6.77	5.73	0.018	6.85	4.94	0.013	6.88	3.53	0.011
^163^Dy	GBW07107	5.1	5.01	5.76	0.008	4.91	5.84	0.016	5.06	3.93	0.003
	GBW07891	21	19.3	8.68	0.037	21.3	3.96	0.006	22.6	2.67	0.032
	GBW07893	30.3	25.6	9.01	0.073	28.3	4.92	0.030	29.7	5.29	0.009
^165^Ho	GBW07107	0.98	0.92	7.51	0.027	0.94	5.17	0.018	0.95	4.76	0.014
	GBW07891	4.04	3.93	4.95	0.012	3.92	3.84	0.013	4.01	1.90	0.003
	GBW07893	4.37	4.21	5.73	0.016	4.29	7.96	0.008	4.46	6.49	0.009
^167^Er	GBW07107	2.7	2.54	5.37	0.027	2.57	3.96	0.021	2.62	4.31	0.013
	GBW07891	11.5	10.6	7.63	0.035	10.9	4.62	0.023	11.1	5.18	0.015
	GBW07893	8.22	8.02	8.16	0.011	8.13	3.58	0.005	8.26	1.69	0.002
^169^Tm	GBW07107	0.43	0.39	7.67	0.042	0.41	4.88	0.021	0.43	4.60	0.000
	GBW07891	1.78	1.64	5.63	0.036	1.70	3.92	0.020	1.76	4.03	0.005
	GBW07893	1.03	0.93	6.81	0.044	1.01	4.95	0.009	1.04	2.69	0.004
^172^Yb	GBW07107	2.6	2.48	4.87	0.021	2.55	3.74	0.008	2.62	1.56	0.003
	GBW07891	11.6	10.7	9.56	0.035	11.1	6.02	0.019	11.4	5.79	0.008
	GBW07893	6.01	5.86	7.61	0.011	5.80	3.55	0.013	6.15	3.67	0.010
^175^Lu	GBW07107	0.41	0.38	5.19	0.033	0.39	4.57	0.022	0.42	3.75	0.010
	GBW07891	1.68	1.57	6.47	0.029	1.66	3.62	0.005	1.69	2.86	0.003
	GBW07893	0.944	0.89	6.05	0.026	0.92	5.24	0.011	0.93	5.31	0.006

It can be seen that the RSD of measured results of closed acid dissolution method for sample treatment ranged from 2.51% to 9.56%, and Δlg*C* ranged from 0.006 to 0.073. The RSD of sample determination results using open acid dissolution with five acids method ranged from 1.93% to 7.96%, and Δlg*C* ranged from 0.004 to 0.045. The RSD of sample determination results processed by sodium peroxide alkali fusion method ranged from 1.24% to 6.49%, and Δlg*C* ranged from 0.001 to 0.032. The results of analyzing standard substance samples using various methods are basically consistent with the recognized values, and the precision and accuracy meet the requirements of specification DZ/T0011-2015. The precision and accuracy of the sample determination results of sodium peroxide alkali fusion method are obviously better than the other two methods, which is the most ideal method for sample analysis in this study.

#### Testing of polymetallic ore samples in Pan-Xi region

In order to confirm the reliability of method, the samples PX-1, PX-2 and PX-3 of polymetallic ore in Pan-Xi region were dissolved according to the above three decomposition methods in this experiment. REE were determined using ICP-MS, and 12 samples were measured in parallel for each polymetallic ore. The relative standard deviation of each element was calculated, and the results were shown in Table 6. It is observed that the results of sample decomposition using sodium peroxide alkali melting method have good consistency with the results of the other two decomposition methods, and RSD is between 0.84% and 4.82%, which is better than the other two methods. This indicates that using sodium peroxide alkali melting method to decompose samples is more suitable for the determination of REE in polymetallic mineral samples in Pan-Xi region by ICP-MS.

**Table 6:** Analytical results of REE in practical samples.

PX-1						
REE	closed acid dissolution method	RSD %	open acid dissolution with five acid method μg.g^-1^	RSD %	alkali fusion with sodium peroxide method μg.g^-1^	RSD %
^89^Y	51.5	4.01	53.4	2.58	53.7	1.83
^139^La	217	2.72	204	3.59	215	2.85
^140^Ce	994	1.99	1058	2.94	1050	2.56
^141^Pr	225	2.83	221	3.02	231	3.24
^146^Nd	521	2.15	527	2.37	540	1.66
^147^Sm	86.1	1.74	83.5	1.28	84.3	0.84
^153^Eu	15.3	1.97	16.5	2.22	16.9	1.82
^157^Gd	19.2	2.88	20.1	1.98	20.6	2.63
^159^Tb	2.95	3.18	3.06	2.74	3.04	3.05
^163^Dy	9.60	4.43	10.5	2.57	10.8	1.36
^165^Ho	1.48	3.76	1.52	3.36	1.57	3.18
^167^Er	3.92	3.62	4.00	2.69	4.06	1.86
^169^Tm	0.86	4.27	0.87	1.90	0.89	1.63
^172^Yb	3.59	3.86	3.68	2.74	3.74	2.02
^175^Lu	0.77	4.94	0.79	3.18	0.83	3.71
PX-2						
^89^Y	83.9	3.46	85.7	2.72	86.4	1.93
^139^La	2688	2.52	2717	1.88	2730	0.93
^140^Ce	3116	3.40	3085	2.67	3160	2.75
^141^Pr	568	2.94	584	3.61	575	3.79
^146^Nd	1255	5.81	1247	4.36	1270	4.78
^147^Sm	164	2.96	156	3.18	162	2.47
^153^Eu	32.0	3.64	31.8	1.77	32.6	0.86
^157^Gd	65.6	3.49	66.3	2.06	67.1	1.63
^159^Tb	6.15	4.70	6.18	3.59	6.23	3.71
^163^Dy	22.8	3.48	22.7	2.83	23.6	2.63
^165^Ho	3.25	2.95	3.28	2.72	3.33	1.56
^167^Er	6.32	4.04	6.43	3.90	6.48	3.78
^169^Tm	1.22	3.77	1.25	2.07	1.24	0.99
^172^Yb	6.33	4.51	6.37	2.26	6.43	2.38
^175^Lu	1.26	4.79	1.27	3.62	1.29	4.03
PX-3						
^89^Y	115	2.88	121	1.95	124	1.74
^139^La	7948	2.03	7970	1.76	8140	0.96
^140^Ce	9660	1.97	9850	2.73	9810	2.36
^141^Pr	1127	3.05	1116	2.53	1130	4.82
^146^Nd	2469	2.17	2481	1.67	2510	1.98
^147^Sm	302	2.73	299	4.62	295	3.14
^153^Eu	53.5	2.58	52.9	1.83	54.6	1.62
^157^Gd	100	4.95	103	2.97	107	3.71
^159^Tb	5.51	3.29	5.68	2.98	5.77	1.84
^163^Dy	28.0	5.37	29.3	1.80	29.8	1.66
^165^Ho	4.63	6.02	4.86	4.77	4.84	2.74
^167^Er	9.8	7.25	10.4	3.61	10.6	2.70
^169^Tm	1.64	6.50	1.68	5.48	1.70	3.75
^172^Yb	7.9	4.96	8.3	2.61	8.6	3.58
^175^Lu	1.48	3.76	1.55	3.87	1.53	1.83

## Conclusion

Aiming at the analysis of REE in light rare earth ore samples in Pan-Xi region, this study compared three sample decomposition methods: closed acid dissolution, open acid dissolution with five acids, and alkali fusion with sodium peroxide. The experimental research found that the detection limit of each rare earth element obtained by closed acid dissolution method was lower than the other two methods. However, when the sample is decomposed by alkali fusion with sodium peroxide method, the minerals can be completely dissolved, and the results of determination of REE are closer to the recognized value. The precision, as well as accuracy, of determination of national rare earth standard substances are significantly better than the other two methods, meeting the requirements of specification DZ/T0011-2015. Through actual verification of polymetallic ore samples in Pan-Xi region, the determination results are accurate and reliable, which can be applied to the rapid analysis of REE in large quantities of polymetallic ore samples from Pan-Xi region.

## Supporting information

S1 Data (XLSX)
